# PRAS40 signaling in tumor

**DOI:** 10.18632/oncotarget.17299

**Published:** 2017-04-20

**Authors:** Dan Lv, Lianying Guo, Ting Zhang, Lin Huang

**Affiliations:** ^1^ Department of Pathophysiology, Dalian Medical University, Dalian, Liaoning 116044, P.R. China

**Keywords:** PRAS40, Akt, mTOR, signaling, tumorigenesis

## Abstract

The proline-rich Akt substrate of 40 kDa (PRAS40) is a substrate of Akt and a component of the mammalian target of rapamycin complex 1 (mTORC1). Locating at the crossroad of the PI3K/Akt pathway and the mTOR pathway, PRAS40 is phosphorylated by growth factors or other stimuli, and regulates the activation of these signaling pathways in turn. PRAS40 plays an important role in metabolic disorders and multiple cancers, and the phosphorylation of PRAS40 is often associated with the tumor progression of melanoma, prostate cancer, etc. PRAS40 promotes tumorigenesis by deregulating cellular proliferation, apoptosis, senescence, metastasis, etc. Herein, we provide an overview on current understandings of PRAS40 signaling in the tumor formation and progression, which suggests that PRAS40 or phospho-PRAS40 could become a novel biomarker and therapeutic target in tumor.

## INTRODUCTION

The proline-rich Akt substrate of 40 kDa (PRAS40) is encoded by *AKT1S1* (Akt1 substrate 1) which locates on the chromosome 19q13.33. 15% of PRAS40's amino acids are prolines, but the function of the two proline-enriched stretches at the N-terminal region remains unknown. PRAS40 was detected in coimmunoprecipitation and far western blot experiments as a 40-kDa protein that bound to 14-3-3 protein in cells treated with insulin [1, 2]. Both mRNA and protein analyses suggest a ubiquitous expression of PRAS40 in multiple tissues of different species [1, 3–5]. PRAS40 shifts between the cytoplasm and the nucleus due to the nuclear export sequence (NES) in the C-terminus [6, 7]. The nuclear PRAS40 contributes to the radioresistence or senescence repression by forming complexes with FOXO3a-14-3-3 [7] or RPL11-HDM2-p53 [8]. The cytoplasmic PRAS40 is involved into the regulation of PI3K/Akt and mTOR pathways. Being an important component of mammalian target of rapamycin (mTOR) complex 1 (mTORC1) [2, 9, 10], PRAS40 regulates multiple functions of mTORC1 [11]. PRAS40 is a substrate of Akt verified both *in vitro* and *in vivo* [1], and mediates the signaling of PI3K/Akt pathway. There are many phosphorylation sites in the C-terminal region of PRAS40, and several phosphorylation sites including Ser183 [12], Ser184 [12], Ser203 [13], Ser212 [14], Ser213 [13], Ser221 [14], Thr246 [1], and Thr247 [12] have been reported to be responsible for growth factor treatments. Most of the reported functions of PRAS40 are related to its phosphorylation [6]. Overexpression or hyperphosphorylation of PRAS40 has been reported in a variety of tumors including melanoma, prostate cancer, gastric cancer, non-small cell lung cancer (NSCLC) and so on [15–18], and plays a critical role in cell survival in different species [5, 15, 19–21]. The high level of phospho-PRAS40 is associated with malignant progression or poor survival of patients [15, 16]. Targeting PRAS40 suppresses tumor growth and induces apoptosis significantly [15, 19, 22]. Here we focus on the signalings of regulation and function of PRAS40 in tumor, which is anticipated to provide a reference for the coming laboratory and clinical studies on PRAS40.

## PRAS40 SIGNALING

### Stimuli

**Figure 1:**
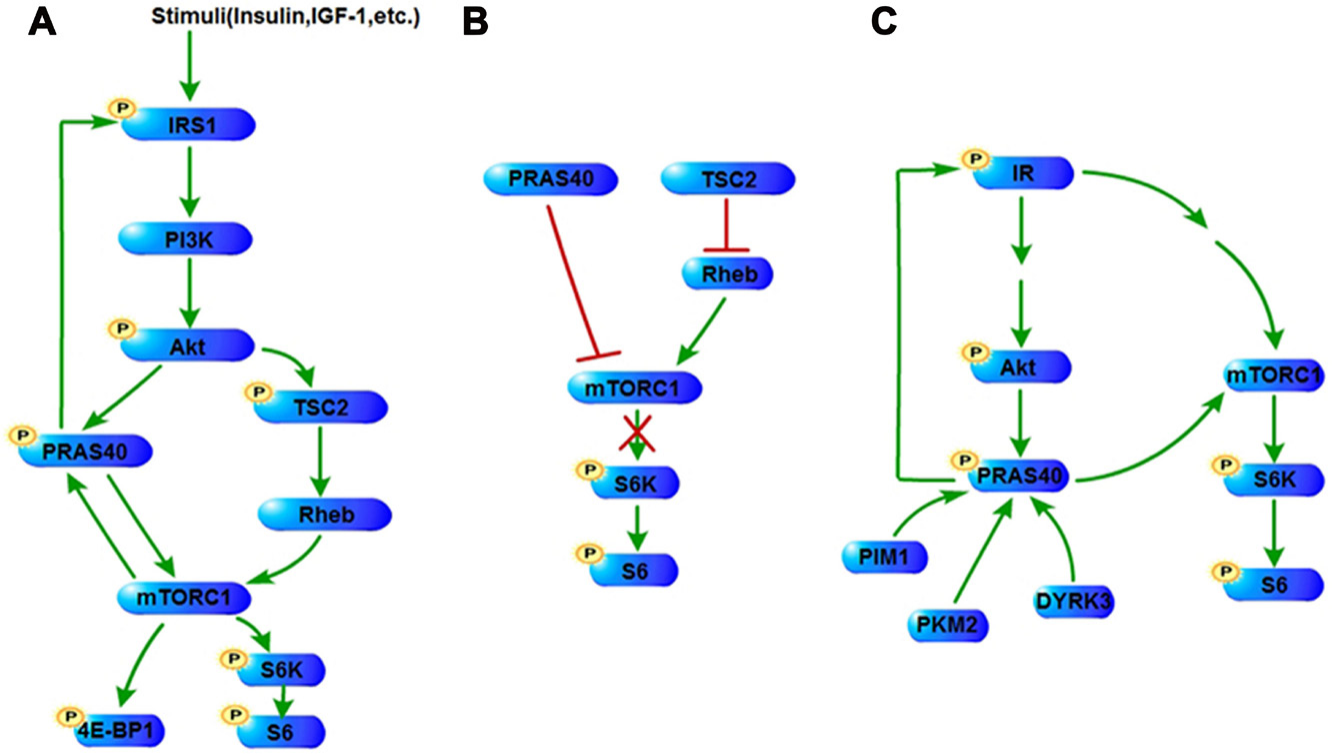
PRAS40 in PI3K/Akt and mTOR pathways (**A**) Stimuli such as insulin and IGF-1 activate PI3K/Akt pathway, resulting in the phosphorylation of PRAS40 and TSC2. Phosphorylation of both PRAS40 and TSC2 reverses their suppression on the activation of mTOR pathway, including the phosphorylation of mTOR's downstream factors, such as 4E-BP1 and S6K. mTORC1 also phosphorylates PRAS40. The phosphorylation of PRAS40 promotes IRS1 expression and phosphorylation in turn through a positive control mechanism. (**B**) Unphosphorylated PRAS40 and TSC2 repress mTORC1 signaling. (**C**) Under certain conditions, phosphorylated PRAS40 promotes the signaling of IR/PI3K/Akt pathway and mTOR pathway through increasing IR phosphorylation. PRAS40 could be either phosphorylated by PIM1, PKM2 and DYRK3. IGF-1, insulin-like growth factor-1; TSC2, tumor sclerosis complex 2; 4E-BP1, eukaryotic translation initiation factor 4E-binding protein 1; S6K, ribosomal protein S6 kinases; IR, insulin receptor; PIM1, Proviral integration site for Moloney murine leukemia virus-1; PKM2, Pyruvate kinase M2; DYRK3, The dual specificity tyrosine-phosphorylation-regulated kinase 3.

Various external stimuli initiate proliferation signaling that promotes tumorigenesis, in which PRAS40 is involved (Figure 1A). Epidemiological evidences indicate that insulin secretion rate and insulin-like growth factor-1 (IGF-1) level influence cancer risk and/or cancer prognosis [23]. Insulin or IGF-1 treatment stimulates the proliferation of tumor cells [24]. PRAS40 is phosphorylated in response to insulin or IGF-1 treatment, and plays an important role in the tumor cell proliferation induced by these growth factors [22, 25]. EGF signaling is hyperactivated in many cancers, such as breast cancer and NSCLC. EGF treatment leads to the PRAS40 phosphorylation that activates mTORC1 [26]. Transforming growth factor-β (TGFβ) signaling exhibits complicated features functioning as either a tumor promoter or a suppressor in cancer biology [27]. TGFβ induces miR-96 expression through Smad-dependent transcription, and miR-96 decreases PRAS40 protein level in prostate cancer cells [28]. In addition, TGFβ stimulates the expression of miR-21 that decreases PTEN expression, resulting in PRAS40 phosphorylation in glomerular mesangial cells [29]. Erythropoietin (EPO) promotes cancer stem cell self-renewal and expansion in an autocrine/paracrine manner, while blocking EPO signaling inhibits tumor growth both *in vitro* and *in vivo* [30]. EPO treatment induces PRAS40 phosphorylation that leads to the interaction of PRAS40 with 14-3-3 and mTORC1 activation [31]. Recently, researches on tumor environment and metabolism show that nutrients including amino acids and glucose are important for tumor proliferation [32, 33]. PRAS40 is barely released from mTORC1 under leucine deprivation compared with leucine supplement [34]. Leucine addition induces the PRAS40 phosphorylation resulting in mTOR activation [35]. High glucose concentration increases PRAS40 phosphorylation in a PI3K/ Akt-dependent manner [36], whereas inhibition of high glucose-induced miR-26a which targets PTEN, blocks phosphorylation of PRAS40, suppressing the phosphorylation of S6 kinase and 4EBP-1 [37].

### PI3K/Akt

The components of PI3K/Akt signaling pathway are frequently altered in tumor, which leads to cell survival, cell cycle progression and cellular proliferation. Activation of phosphatidylinositol 3-kinase (PI3K) induces the conversion of phosphatidylinositol 4,5-bisphosphate (PIP2) to phosphatidylinositol-3,4,5-triphosphate (PIP3) and the consequent activation of phosphoinositide-dependent kinase 1 (PDK1) and Akt. Activated Akt is able to phosphorylate PRAS40 on Thr246 either *in vivo* or *in vitro*, which leads to the binding to 14-3-3 [1]. Mouse embryo fibroblasts (MEFs) lacking Akt1 and Akt2 exhibit a diminished level of phosphorylated PRAS40 [1]. Treatment with Akt or PI3K inhibitor suppresses the phosphorylation of PRAS40 [3, 38]. The phosphorylation of Akt and PRAS40 induced by IGF-1 in PC12 cells was inhibited by the PI3K specific inhibitor, while no inhibitory effect was observed under the treatments of ERK and p38 inhibitors [39]. mTORC2 phosphorylates Akt on Ser473 which is responsible for PRAS40 phosphorylation. A partial inhibition of the PRAS40-Thr246 phosphorylation has been observed with the treatment suppressing mTORC2 activity in a cell type-dependent manner [40–42]. Therefore PRAS40 is a substrate of Akt and could be phosphorylated by the activated PI3K/Akt pathway (Figure 1A).

PRAS40 augments the activation of PI3K/Akt pathway (Figure 1). Gene transfer of PRAS40 reduces the infarction size in rat brains and promotes Akt phosphorylation [43]. Accordingly, Akt phosphorylation is decreased in the liver tissues of PRAS40 knockout mice [44]. Furthermore, a notable increase of Akt phosphorylation is induced by PRAS40 overexpression in Ewing sarcoma family tumor (ESFT) cells [22], whereas a significant decrease of Akt phosphorylation with PRAS40 knockdown has been found in ESFT cells, liver cancer cells, skeletal muscle cells and fibroblasts [22, 34, 44, 45]. The influence of PRAS40 on the activation of the upstream factors suggests that PRAS40 controls a positive feedback in the insulin receptor/PI3K/Akt pathway. On the contrary, Fonseca B D et al. reported that no remarkable change was observed on Akt phosphorylation when PRAS40 was knocked down in HEK293 and Hela cells [2].

For the mechanism through which PRAS40 stimulates PI3K/Akt signaling, the roles of the upstream factors have been indicated. IRS1 expression decreases in PRAS40 T246A expressing or PRAS40-knockdown 3T3-L1 cells [34], and IRS1 degradation is promoted by increased proteasome activity in PRAS40-knockdown myotubes [45] (Figure 1A). In addition, our data show that the phosphorylation of insulin receptor is remarkably increased by PRAS40 overexpression whereas decreased by PRAS40 deletion [22] (Figure 1C).

### PIM1

Although the Thr246-phosphorylation of PRAS40 is mainly induced by Akt, there is also Akt-independent mechanism. The Thr246-phosphorylation of PRAS40 stimulated by insulin treatment decreases but remaines at a lower level in the heart cells of PDK1 knockout mice [35]. An Akt kinase specific inhibitor attenuates the IGF-1-induced PRAS40-Thr246 phosphorylation in PC12 cells only at higher concentration. However, the PRAS40-Thr246 phosphorylation cannot be completely abolished by the treatment of Akt inhibitors [39].

Proviral integration site for Moloney murine leukemia virus-1 (PIM1) plays an essential role in the regulation of cell proliferation, cell survival and multiple drug resistance. Zhang F et al. have reported that PIM1 overexpression increases the Thr246-phosphorylation of PRAS40 independently of Akt activation in mouse bone marrow cells [46]. PIM1 is capable to directly phosphorylate PRAS40 on Thr246 *in vitro* and *in vivo* [7, 46] (Figure 1C). In cancer cells, resistance to ionizing radiation appears to be a big problem in the clinical setting of lung cancer treatment. In radio-resistant cells, exposure to radiation leads to the overexpression and the nuclear translocation of PIM1. Increased nuclear PIM1 phosphorylates PRAS40, resulting in the formation of a trimeric complex of phospho-PRAS40, 14-3-3 and Akt-phosphorylated FOXO3a, which was detected by coimmunoprecipitation. The resulting complex moves to cytoplasm consequently and the cytoplasmic retention of FOXO3a is associated with the decrease of proapoptotic genes and radioresistance [7].

### PKM2 and DYRK3

Pyruvate kinase M2 (PKM2) binds and phosphorylates PRAS40 *in vivo* and *in vitro*. Ser202/203-phosphorylation of PRAS40 by PKM2 releases PRAS40 from mTORC1 and facilitates its binding to 14-3-3, which results in stimulations-independent activation of mTORC1 signaling for oncogenic growth and autophagy inhibition in cancer cells [47] (Figure 1C).

The dual specificity tyrosine-phosphorylation-regulated kinase 3 (DYRK3) is able to couple the stress granules (SGs) formation and mTORC1 signaling. Inactive DYRK3 prevents SG dissolution and sequesters mTORC1 into SG during stress. Exposure to stimuli, active DYRK3 allows SG dissolution releasing mTORC1, and phosphorylates PRAS40 directly *in vivo* and *in vitro* that activates mTORC1 signaling [26] (Figure 1C).

### mTOR

mTOR regulates cellular growth and metabolism through two structurally and functionally distinct complexes, mTORC1 and mTORC2. Besides mTOR, mTORC1 contains regulatory-associated protein of mTOR (raptor), mammalian lethal with Sec13 protein 8 (mLST8) /G-protein β-subunit-like protein (GβL), DEP-domain-containing partner of mTOR (deptor), while mTORC2 contains rapamycin-insensitive companion of mTOR (rictor), mammalian stress-activated protein kinase interacting protein (mSin1), mLST8/GβL, protein observed with rictor-1 (protor-1), and deptor [11]. PRAS40 preferentially binds to raptor *in vitro* and *in vivo* [2, 20, 34, 48], but does not present in rictor immunoprecipitates, suggesting that PRAS40 is a component of mTORC1, but not of mTORC2 [10, 14, 48]. mTORC1 signaling is repressed by the tumor sclerosis complex 2 (TSC2) which is phosphorylated and inhibited by Akt. Activated mTORC1 mediates the phosphorylation of eukaryotic translation initiation factor 4E-binding protein 1 (4E-BP1) and ribosomal protein S6 kinases (S6K), resulting in the release of eukaryotic translation initiation factor 4E (eIF4E) and phosphorylation of S6 [1, 10] (Figure 1A).

In response to the treatment of growth factors, PRAS40 is phosphorylated on Thr246 by Akt, and phosphorylated on Ser183 and Ser221 by mTOR (Figure 1A), all of which are essential to the dissociation of PRAS40 from the mTORC1 and the binding of PRAS40 to the cellular anchor protein 14-3-3 in HEK293 cells and fibroblasts [1, 2, 34, 49, 50]. The transfection of a dephosphomimic mutant form of PRAS40, PRAS40 (T246A), or the Akt inhibitor treatment, represses the Ser183-phosphorylation of PRAS40 in HEK293 cells, suggesting that the Thr246-phosphorylation facilitates the Ser183-phosphorylation of PRAS40 [34, 49]. However, either PRAS40 (T246A) transfection, or the treatment of rapamycin or Akt inhibitor does not alter the Ser183-phosphorylation of PRAS40 in ESFT cells, suggesting that it is not mTOR that phosphorylates PRAS40 on Ser183 in ESFT cells [22]. Therefore, the cellular context should be important to induce the Ser183-phosphorylation of PRAS40.

PRAS40 phosphorylation promotes the activation of mTOR signaling pathway (Figure 1). Gene transfer of PRAS40 reduces the infarction size in rat brains and promotes mTOR phosphorylation [43]. PRAS40 increases the activation of mTOR pathway in ESFT cells and glomerular mesangial cells [22, 29, 36, 37]. Transgenic expression of PRAS40 (T246A) in basal keratinocytes of mouse epidermis results in an inactivation of the mTOR pathway [51]. PRAS40 dephosphorylation is associated with the inhibition of mTOR activity in renal cancer cells [25]. In accordance with these reports, the activation of mTOR pathway is downregulated in ischemic brains of PRAS40 knockout mice [43]. Silencing of PRAS40 by small interfering RNA suppresses the mTOR pathway signaling in response to insulin treatment in HEK293 cells, adipocytes, liver cancer cells and ESFT cells [2, 14, 22, 34].

However, the effects of PRAS40 on mTOR pathway remain controversial. Vander Haar et al. found that the mTOR pathway signaling was activated instead with PRAS40 knockdown in the adipocytes and the liver cancer cells cultured in media containing serum contrast to that in the cells stimulated by insulin [34]. Mi B et al. found that mTOR kinase activity was increased by PRAS40 knockdown in breast cancer cells and colon cancer cells [52]. Sancak Y et al. reported PRAS40 inhibited the mTOR kinase activity *in vitro* and in the HEK293 cells cultured in media containing serum [10]. Wang L et al. and Forseca B D et al. also reported that PRAS40 overexpression inhibited the mTOR kinase activity in HEK293 cells although PRAS40 knockdown also inhibited the mTOR kinase activity in the same cells [2, 14, 39]. Malla R et al. reported that mTOR activation was increased in the liver tissues of PRAS40 knockout mice [44] (Figure 1A). However, PRAS40 knockdown does not make any alteration on mTOR pathway in myoblasts [53].

Therefore, the influence of PRAS40 on the signaling of mTOR pathway may be controlled by several factors. At first, PRAS40 phosphorylation on different sites may play different roles on mTOR activation, due to the contrast responses of the same cells cultured in the media containing serum or insulin [2, 10, 14, 34]. Next, since PRAS40 overexpression and knockdown showed either increased or decreased mTOR activity [2, 14, 22, 29, 34, 36, 37], while the different transfection efficiency could result in different PRAS40 levels, the ratio of phospho-PRAS40 v.s. unphospho-PRAS40 may be important. Further, the different mTOR activity in brain tissues and liver tissues of PRAS40 knockout mice reported by two labs suggests a tissue specific manner [43, 44]. More studies are required to clarify the relationship of PRAS40 and mTOR in detail.

## PRAS40 IN TUMOR

### Proliferation

The Akt3 signaling pathway is deregulated in about 70% of advanced stage melanoma [54, 55]. Majority of melanoma samples with elevated Akt activity also show corresponding higher levels of PRAS40 phosphorylation, and siRNA-mediated inhibition of Akt3 reduces the levels of PRAS40 phosphorylation. Targeting PRAS40 reduces the anchorage-independent growth of melanoma cells in culture significantly and inhibited the tumor development of xenograft. Reduction of total or phosphorylated PRAS40 increases the apoptosis of melanoma cells [15]. PRAS40 expression level increases in ESFT cells, PRAS40 silencing suppresses the proliferation of ESFT cells [10, 19, 22]. Loss of PTEN is the most common genetic alteration observed in prostate cancer, resulting in an increase of PI3K/Akt activation. The upregulation of PRAS40 phosphorylation has been found in prostate cancer and phospho-PRAS40 is indicated as a biomarker for prostate cancer [16, 56]. The abnormal activation of PI3K/Akt is often found in NSCLC patients, and the level of PRAS40 phosphorylation also increases in NSCLC [17]. PRAS40 phosphorylation is increased by recombinant IL-15 treatment in the breast cancer cells with high level of IL-15RA, which promotes cell proliferation [57]. Elevated IGF-1 signaling in renal cell carcinoma (RCC) patients is associated with poor prognosis, and correlates with the potency of tumor development and progression. IGF-1 induces PRAS40 phosphorylation in an Akt-dependent manner, since PRAS40 (T246A) significantly attenuates the IGF-1R-driven proliferation of renal cancer cells [25]. PRAS40 (T246A) transgenic mice have less response to 12-O-tetradecanoylphorbol-13-acetate (TPA)-induced epidermal hyperproliferation and skin tumor promotion [51]. The data in myoblasts show that PRAS40 knockdown results in a greater proportion of cells in G1/G0 phase of the cell cycle and fewer cells in the active S phase compared with control values [53]. Abnormal expression or activation of PRAS40 in tumor may promote the cellular proliferation by deregulating cell cycle.

### Apoptosis

Gene transfer of PRAS40 reduces infarction size of cerebral ischemia in rats by promoting the phosphorylation of Akt, FKHR (FOXO1), mTOR. In contrast, the infarction size is increased in PRAS40 knockout mice [43]. H_2_O_2_ mediated hypoxia induces the phosphorylation of PRAS40 resulting in a protection for brain cells against apoptosis [58, 59]. Human amniotic fluma stem (AFS) cells have high proliferation potential but a lower risk for tumor development. PRAS40 knockdown leads to massive apoptotic cell death during embryoid bodies (EBs) development of human AFS cells, which was found to be dependent on mTOR [60]. PRAS40 knockdown induced by siRNAs increases the expression level of cleaved caspase 3 and the rates of tumor cell apoptosis in melanoma and ESFT cells [15, 19, 22]. P2×7 receptor is involved in immune modulation and cell survival. Extracellular adenosine triphosphate (ATP) is able to activate P2×7 receptor to induce cell death efficiently. The dephosphorylation of PRAS40 and mTOR pathway by AMPK was found to be responsible for the apoptosis led by ATP-P2×7 signaling [61]. The phosphorylation of PRAS40 is downregulated by MYO6 knockdown which inhibits the growth and induces the apoptosis of prostate cancer cells [62]. Taken together, the anti-apoptotic role of PRAS40 is dependent on the suppression of mTOR signaling. However, Kathrin Thedieck's study stated that PRAS40 deficiency prevented the induction of apoptosis by TNF-α and cycloheximide in Hela cells, which could not be mimicked by rapamycin treatment [48], suggesting an unexpected pro-apoptotic role of PRAS40 independently of mTOR.

### Senescence

**Figure 2:**
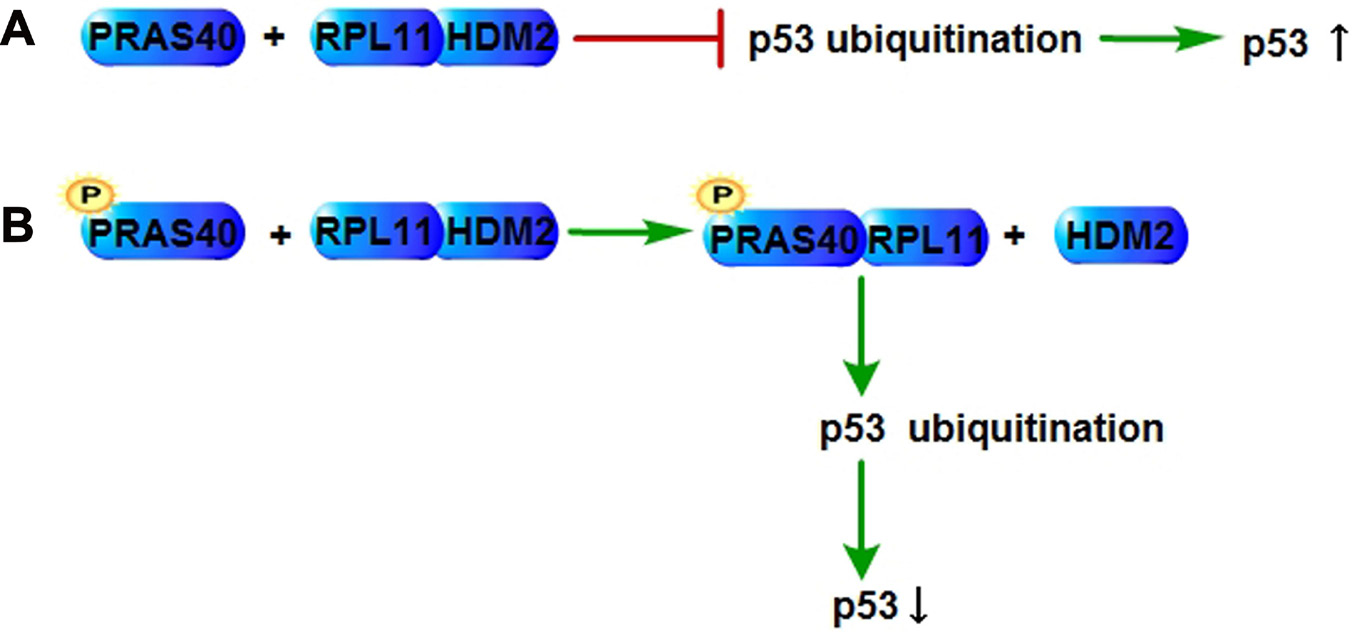
PRAS40 in p53 regulation (**A**) RPL11 interacts with HDM2, repressing the ubiquitination of p53, which increases the expression level of p53. (**B**) The interaction of RPL11 and HDM2 could be inhibited by the competitive binding of phospho-PRAS40 and RPL11, which results in the p53 ubiquitination and a decrease of the expression level of p53. RPL11, Ribosomal protein L11; HDM2, Human Double Minute 2.

The tumor suppressor p53 controls several cellular biological actions including apoptosis, senescence, genomic stability, etc. The ribosomal protein (RP)- Human Double Minute 2 (HDM2)-p53 pathway plays key roles in apoptosis and senescence through controlling p53 expression level. Ribosomal protein L11 (RPL11) binds to the acidic region of HDM2, which inhibits the ubiquitination of p53 that upregulates the p53 expression in response to stress. The phosphorylation of PRAS40 induced by growth factors or nutrients promotes the nuclear-specific association of PRAS40 with RPL11, which was detected by coimmunoprecipitation, resulting in a decrease of the RPL11-HDM2 complex level as well as the p53 expression level. Silencing of PRAS40 increases the p53 expression level in a RPL11-dependent manner, which can be rescued by wild type PRAS40, but not by the RPL11-binding-null PRAS40 (T246A) mutant. Therefore, PRAS40 negatively regulates the RPL11-HDM2-p53 nucleolar stress response pathway through the competitive interaction with RPL11 and suppresses the p53-mediated cellular senescence [8] (Figure 2).

Mitogen-activated protein kinase phosphatase-7 (MKP7) acts as a negative regulator of MAPK in mammalian cells [63]. MKP7 Overexpression in MEFs decreases greatly the phosphorylation levels of mTOR and PRAS40, whereas MKP7 deletion restores their phosphorylation. MKP7 represses cellular senescence by dephosphorylating mTOR and PRAS40, and forming complexes with them, which was clarified by coimmunoprecipitation [64].

### Metastasis

**Figure 3:**
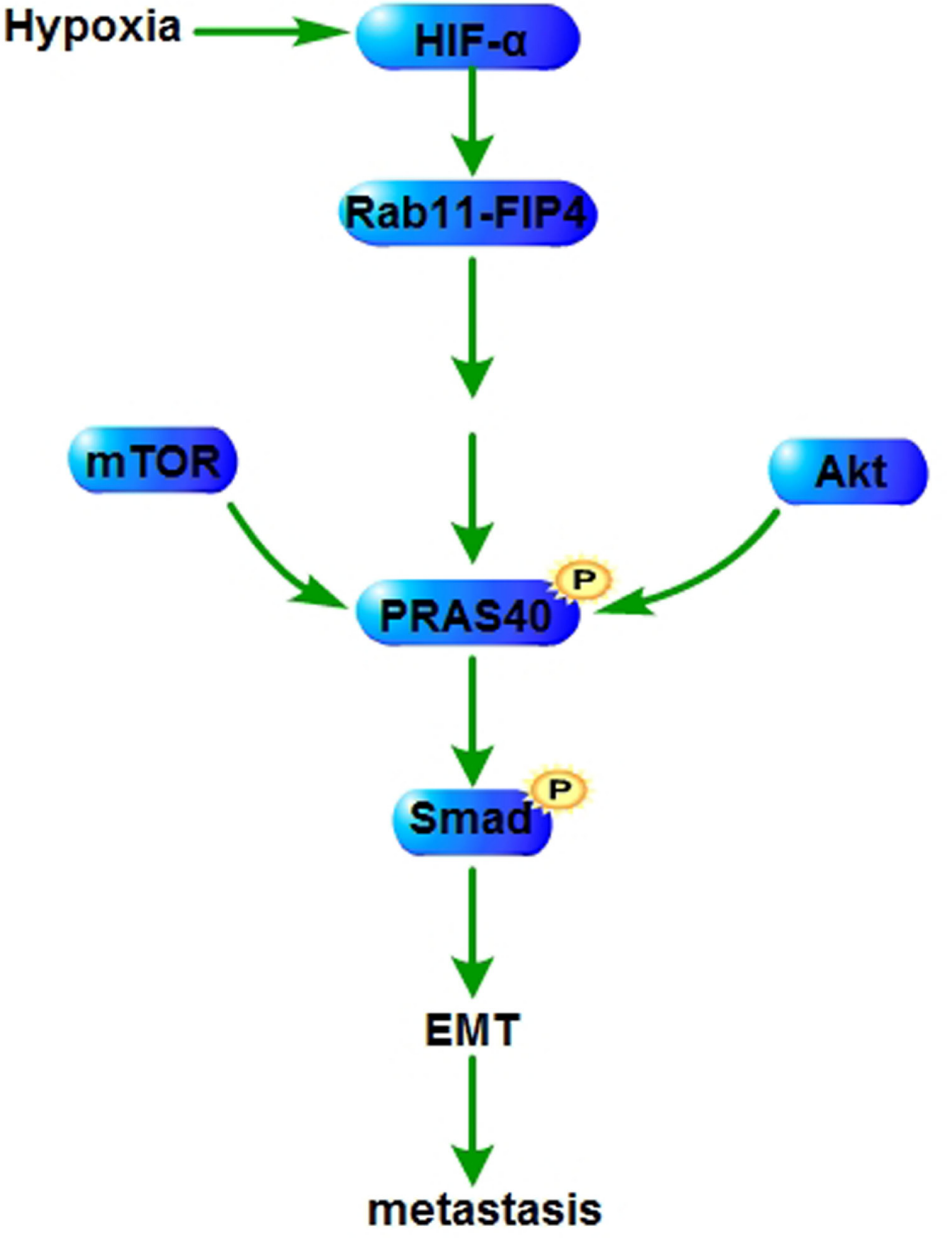
PRAS40 in metastasis HIF-1α induced by hypoxia augments the expression of Rab11-FIP4 which enhances PRAS40 phosphorylation. Phospho-PRAS40 increases the expression and the activation of EMT related factors to promote metstasis through amplifying the phosphorylation of smad. HIF-1α, Hypoxia inducible factor 1alpha; EMT, epithelial-mesenchymal transition; Rab11-FIP4, Rab11-family interacting protein.

Hypoxia inducible factor 1alpha (HIF-1α) plays a critical role in the response of tumors to hypoxia, and contributes to tumor aggression, invasion and resistance to radiotherapy and chemotherapy [65]. Hypoxia induces the expression of Rab11-family interacting protein 4 (Rab11-FIP4) through the transcriptional regulation of HIF-1α. Rab11-FIP4 overexpression upregulates PRAS40 phosphorylation, increasing the migration and invasion of hepatocellular carcinoma (HCC) cells. PRAS40 deletion or dephosphorylation suppresses the migration and invasion of HCC cells induced by Rab11-FIP4 overexpression in the mTOR-dependent manner [66]. PRAS40 deletion also decreases the motile and invasive ability of ESFT cells [19]. PRAS40 (T246A) transgenic mice show a decreased keratinocyte migration which is correlated with altered expression of epithelial-mesenchymal transition (EMT) markers and reduced smad signaling [51] (Figure 3).

### Immunoregulation

mTOR regulates the functional outcome of diverse immune cells, including T cells, B cells, dendritic cells, macrophages, neutrophils, mast cells and natural killer cells [67]. Activated mTORC1 accelerates T-cell infiltration at the tumor site and exerts more aggressive immune pressure on the growing tumors, which is associated with the prevention of tumor escape. Overexpression of PRAS40 in T cells decreases the mTORC1 signaling, promotes the T cell quiescence and blocks the tumor infiltration of the T cells *in vitro* and *in vivo* [68].

### Protein degradation

Protein stress, such as misfolded proteins or oxidized proteins aggregate, occurs in many diseases, including cancer. Hyperactivation of mTORC1 reduces translational fidelity as a result of increasing the rates of ribosomal elongation [69]. Recently, mTORC1 was reported to be able to promote the assembly of immunoproteasomes via PRAS40 to adapt cells to protein stress. Immunoproteasomes are highly expressed in immune cells but also present in other cells, the proteolytic β-subunits of which are β1i, β2i and β5i instead of β1, β2 and β5 in constitutive proteasomes. Using coimmunoprecipitation, the authors show that PRAS40 binds to the immunoproteasome β-subunit (iβ) precursors and suppresses the immunoproteasome formation. Phosphorylation on Ser183 and Ser221 by mTORC1 promotes the dissociation of PRAS40 from iβ precursors, resulting in the maturation of iβs and the immunoproteasome formation. Therefore mTORC1 phosphorylates PRAS40 to enhance protein synthesis and simultaneously to facilitate the assembly of the β subunits for forming immunoproteasomes [70]. Consistent with this report, PRAS40 deletion decreases protein synthesis in C2C12 myoblasts [53].

## CONCLUSIONS AND FUTURE PERSPECTIVES

Similar as other adaptor proteins, such as Grb2 and Crk, PRAS40 binds to a couple of factors and links several important signaling pathways especially PI3K/Akt and mTOR pathways. As a consequence of these interactions, PRAS40 can regulate and integrate the signaling events, providing spatiotemporal control and specificity, therefore promotes proliferation, represses apoptosis, decreases senescence, increases metastasis, enhances protein degradation and limits immune response. PRAS40 is believed to contribute to tumorigenesis, and PRAS40 or phospho-PRAS40 could become a novel biomarker or therapeutic target in tumor.

However, the regulation of PRAS40 activity remains to be clarified. Phosphorylation is considered as the most important regulation. Although the different roles of phospho- and unphospho-PRAS40 in cells are largely unknown, some of the functions are found to be accomplished by the phosphorylation form. Under insulin treatment, PRAS40 phosphorylation is induced, meanwhile the interaction of PRAS40 and mTORC1 is decreased whereas that of PRAS40 and 14-3-3 is increased [1]; IRS1 expression is decreased in PRAS40 T246A expressing 3T3-L1 cells, resulting in the inactivation of PI3K/Akt signaling [34]; FOXO3a-14-3-3 presents in the immunoprecipitates of phospho-PRAS40 but not unphospho-PRAS40 [7]; phospho-PRAS40 but not unphospho-PRAS40 binds RPL11, leading to a decrease of the RPL11-HDM2 complex level as well as the p53 expression level [8]; unphospho-PRAS40 binds to immunoproteasome subunits and suppresses immunoproteasome formation, whereas phospho-PRAS40 dissociates from immunoproteasome subunits and promotes immunoproteasome formation [70]. Although most studies show that PRAS40 is phosphorylated by the activated PI3K/Akt pathway or mTOR pathway, PIM1, PKM2 and DYRK3 are also confirmed kinases which are responsible for the Thr246-phosphorylation of PRAS40. Whether other pathways are possible to induce PRAS40 phosphorylation remains as an interesting topic.

Although there is not any chemical reported to target either the expression or the phosphorylation of PRAS40 directly, the chemicals lead to PRAS40 dephosphorylation showed antitumor activity. An antitumor chemical, Benzyl Isothiocynate (BITC), decreases PRAS40 phosphorylation and increases the stability of p53. Constitutively active PRAS40 reverses the BITC-induced increase of p53 protein level [71]. Both Akt and mTOR inhibitors suppress tumor proliferation and PRAS40 phosphorylation, while combination of Akt inhibitor MK2206 and mTOR inhibitor rapamycin exhibits profound antitumor effects by suppressing both Thr246 and Ser183 phosphorylation of PRAS40 [52]. Targeting the interaction of PRAS40 and its binding partners could be another anticipated way to inhibit tumor proliferation.

Furthermore, the working model of PRAS40 is yet to be determined. PRAS40 has been reported to regulate the activation of PI3K/Akt and mTOR pathways in a context-dependent manner. Analyzing the altered signaling pathways with PRAS40 overexpression or deletion may help to clarify the other downstream pathways of PRAS40. The protein composition of the PRAS40-containing complex is not fixed, but dynamically regulated. Accumulated or phosphorylated PRAS40 may promote carcinogenesis by cooperating with the binding partners. The elucidation of the entire protein network induced by the abnormal PRAS40 may provide new insights into the molecular mechanism of PRAS40 function.

## Abbreviations

PRAS40: The proline-rich Akt substrate of 40 kDa
mTOR: the mammalian target of rapamycin
PI3K: phosphatidylinositol 3-kinase
PDK1: phosphoinositide-dependent kinase 1
IGF-1: insulin-like growth factor-1
ERK: Extracellular-signal-regulated kinases
PIM1: Proviral integration site for Moloney murine leukemia virus-1
4E-BP1: eukaryotic translation initiation factor 4E-binding protein 1
S6K: ribosomal protein S6 kinases
TSC2: tumor sclerosis complex 2
PTEN: Phosphatase and tensin homolog
IL-15RA: IL-15 receptor alpha
TPA: 12-O-tetradecanoylphorbol-13-acetate
RPL11: Ribosomal protein L11
HDM2: Human Double Minute 2
MKP-7: Mitogen-activated protein kinase phosphatase-7
HIF-1α: Hypoxia inducible factor 1alpha
Rab11-FIP4: Rab11-family interacting protein
EMT: epithelial-mesenchymal transition
NSCLC: non-small cell lung cancer
ESFT: Ewing sarcoma family tumor
MEF: Mouse embryo fibroblast
RCC: renal cell carcinoma
HCC: hepatocellular carcinoma
